# Physiological increases in lactate inhibit intracellular calcium transients, acidify myocytes and decrease force in term pregnant rat myometrium

**DOI:** 10.1113/JP270631

**Published:** 2015-09-03

**Authors:** Jacqui‐Ann Hanley, Andrew Weeks, Susan Wray

**Affiliations:** ^1^ Departments of Cellular and Molecular Physiology; ^2^ Women's and Children's HealthInstitute of Translational Medicine, University of LiverpoolLiverpoolUK

## Abstract

**Key points:**

- Lactate is increased in myometrial capillary blood from women in slow or non‐progressive labour (dystocia), suggesting that it is detrimental to uterine contractions.
- There are no studies of the effect of lactate on the myometrium. In the present study, we have investigated its effects and mechanism of action on myometrial strips from term pregnant rats.
- We show that lactate significantly decreased spontaneous contractility. Lactatedecreased pH_i_ and inhibited Ca^2+^ transients. Nulling the decrease in pH_i_ abolished the effects of lactate effects. If Ca^2+^ entry was enhanced, the effects of lactate were abolished.
- The present study suggests that the accumulation of extracellular lactate could contribute to labour dystocia.

**Abstract:**

Lactate is increased in myometrial capillary blood from women in slow or non‐progressive labour (dystocia), suggesting that it is detrimental to uterine contractions. There are, however, no studies of the effect of lactate on the myometrium. We therefore investigated its effects and mechanism of action on myometrial strips from term pregnant rats. The effects on spontaneous and oxytocin‐induced contractility in response to sodium lactate and other weak acids (1–20 mM) were studied. In some experiments, simultaneous force and intracellular Ca^2+^ or pH (pH_i_) were measured with Indo‐1 or Carboxy‐SNARF, respectively. Statistical differences were tested using non‐parametric tests. Lactate significantly decreased spontaneous contractility with an EC_50_ of 3.9 mM. Propionate, butyrate and pyruvate also reduced contractions with similar potency. The effects of lactate were reduced in the presence of oxytocin but remained significant. Lactate decreased pH_i_ and nulling the decrease in pH_i_ abolished its effects. We also show that lactate inhibited Ca^2+^ transients, with these changes mirroring those produced on force. If Ca^2+^ entry was enhanced by depolarization (high KCl) or applying the Ca^2+^ channel agonist, Bay K 4644, the effects of lactate were abolished. Taken together, these data show that lactate in the physiological range potently decreases myometrial contractility as a result of its inhibition of Ca^2+^ transients, which can be attributed to the induced acidification. The present study suggests that the accumulation of extracellular lactate will reduce myometrial contractions and could therefore contribute to labour dystocia.

**Abbreviations**
AUC: area under the curve
pH_i_: intracellular pH
PMCA: plasma membrane Ca^2+^‐ATPase
PSS: physiological saline solution

## Introduction

For labour to successfully end in normal delivery, the uterus needs to produce strong co‐ordinated contractions. The processes and pathways that achieve this have been characterized somewhat (Wray, 2007), although uncertainties remain, particularly with regard to human labour (Young, 2007). However, when contractions are weak and unco‐ordinated, labour slows and often cannot progress successfully. This is termed dysfunctional or dystocic labour and is a significant cause of non‐elective (emergency) Caesarean sections and is associated with increased morbidity for mother and baby (Saunders *et al*. 1992; Shields *et al*. 2007). The physiological reasons for poor contractions in such labours are not known.

We have previously shown that there is significantly increased lactate in myometrial capillary blood from women suffering dysfunctional labour and also that this blood is of a reduced pH (Quenby *et al*. 2004). Other recent work has reported an increase in lactate in the amniotic fluid of women suffering dysfunctional labour (Wiberg‐Itzel *et al*. 2008; Wiberg‐Itzel *et al*. 2014). In vascular smooth muscle, lactate can cause vasodilatation and depressed responsiveness to agonists (Barron & Nair, 2003). Thus, if similar effects occur in the myometrium, increased lactate may be a significant cause of dysfunctional labour.

Lactic acid is produced by glycolysis in all cells and is dissociated into lactate and protons at physiological pH (Philp *et al*. 2005). Glycolysis mainly occurs under hypoxic conditions, although the uterus is highly glycolytic even under normoxic conditions (Wray, 1990; Steingrimsdottir *et al*. 1995). Lactate has to be transported out of the cell and intracellular pH (pH_i_) maintained within normal limits, and this is mediated by a family of proton‐linked monocarboxylate transporters (Kirk *et al*. 2000; Bonen, 2001). In the myometrium, pH changes occur during each contraction in labour as a result of vascular occlusion at the peak of contraction (Wray *et al*. 1992; Larcombe‐McDouall *et al*. 1999) and it has already been shown that intracellular acidification can decrease uterine contractility (Crichton *et al*. 1993; Parratt *et al*. 1994).

Intracellular acidification inhibits L‐type Ca^2+^ channels in myometrial cells (Shmigol *et al*. 1995) and causes membrane hyperpolarization (Taggart *et al*. 1996), thereby decreasing the force of contraction. Although it is anticipated that lactate will decrease pH_i_, this has not been studied to any great extent in smooth muscle and there are no measurements in the myometrium. Any weak acid such as lactate should acidify tissues. In the myometrium, one such acid, butyrate, has been shown to reduce pH and force (Wray *et al*. 1992). Thus, the question arises as to whether the effects of lactate may be attributed to its effect on pH_i_ or whether it has additional mechanisms of action, as suggested for vascular smooth muscle (Hester *et al*. 1980).

Changes of pH_i_ have been shown to change intracellular Ca^2+^ in the myometrium, with acidification reducing Ca^2+^ transients (Taggart *et al*. 1996; Pierce *et al*. 2003). Thus, a fall in Ca^2+^ might be anticipated when lactate rises, although there are no measurements of intracellular Ca^2+^ under these conditions. During labour, contractions are augmented by oxytocin. The ability of oxytocin to increase membrane excitability and intracellular Ca^2+^ (Parkington *et al*. 1999; Arrowsmith & Wray, 2014) may therefore be anticipated to mitigate the effects of increased lactate, (Taggart *et al*. 1996), although this hypothesis has not been tested. Without such data, it is hard to understand how lactate is acting on the myometrium and what its role in myometrial physiology and pathophysiology may be.

In summary, there are no data available to help clarify the functional effect of lactate on myometrial contractility or its mechanism of action. We hypothesized that an increased lactate level in the myometrium will alter contractility, although these effects will be mitigated by oxytocin. The present study therefore aimed to:

- investigate the effect of lactate on spontaneous contractile activity in ratmyometrium;
- compare the effect of lactate on spontaneous and oxytocin‐driven contractions;
- determine the effects of other weak acids on spontaneous and oxytocin‐driven contractions; and
- investigate the mechanism of the actions of lactate by determining the changes that it elicits in myometrial pH_i_ and Ca^2+^ signalling.

## Methods

### Tissue

Pregnant Wistar rats (Wistar, Charles River, United Kingdom) at 22 days of gestation were humanly killed using CO_2_ anaesthesia and cervical dislocation. The uterus was removed, cleaned and longitudinal myometrial strips (4 mm × 1 mm) were dissected. Individual strips were mounted between a fixed support and a force transducer using aluminium clips in a 1 ml bath and were continuously superfused with physiological saline solution (PSS) (pH 7.40) at a rate of 2 ml min^−1^ and maintained at 35 °C (Babiychuk *et al*. 2004).

### Calcium and pH measurements

For measurement of pH_i_ or Ca^2+^, myometrial strips were loaded with fluorescence indicators Carboxy‐SNARF AM or Indo‐1 AM, respectively (15 μm; 3.5 hours at room temperature), as described previously (Burdyga *et al*. 1995). After loading, the strips were washed for 30 minutes in PSS and mounted in a small bath. Carboxy‐SNARF loaded strips were excited at 530 nm using a xenon lamp and emitted light was detected by photomultipliers at 590 and 650 nm (Taggart & Wray, 1993). For Indo‐1 AM loaded tissue, the wavelengths were 350 nm (excitation) and 400 and 500 nm (emission). Both were digitally recorded at a sampling rate of 100 Hz and the ratio of these two signals was used to report changes in Ca^2+^ or pH. The pH_i_ recordings were calibrated using the K^+^ /H^+^ ionophore nigericin (Austin & Wray, 1993).

### Contraction measurements

A 40 mM high potassium (KCl, 1 min) solution was used to test tissue viability and measure maximal force contraction. Contractile activity in rat myometrial strips was observed for ∼30 minutes after the application of KCl until steady contractions appeared. Once stable contractions were established, contractility was determined during exposure to PSS containing sodium lactate (1–20 mm, pH 7.40) or other sodium salts of weak acids: butyrate, propionate and pyruvate. Previous experiments established that the addition of up to 20 mm NaCl to PSS has no effect on contractility (Kupittayanant *et al*. 2001) and this was reconfirmed in the present study (*n* = 3; data not shown).

### Solutions

Buffered PSS composed of (mm): 154 NaCl, 5.6 KCL, 1.2 MgSO_4_, 2 CaCl_2_, 8 glucose and 10.2 Hepes. KCl solution (40 mM) was made by isosmotically substituting for NaCl after the KCl contraction the strips were returned to PSS. Oxytocin was added to PSS at a final concentration of 0.1 nm. The Ca^2+^ channel agonist Bay K8644 (0.1 μm) was used in some experiments. All chemicals were obtained from Sigma (Poole, UK), unless otherwise stated. Carboxy‐SNARF and Indo‐1 AM were obtained from Molecular Probes (Carlsbad, CA, USA).

### Analysis

Data were analysed using Origin, version 8.2 (MicroCal, Inc., Northampton, MA, USA). The amplitude and duration (measured at 50% of maximal value) of each contraction was measured and the average in the last 2 minutes of solution application was used. Frequency was measured as the number of contractions during lactate application.The area under the curve (AUC; integral of force) was measured during the last 2 minutes of solution application or control period and is presented as a percentage of the control period (100%). Statistical differences were tested using non‐parametric statistical tests (as detailed in text) and *t* tests in SPSS, version 20 (IBM Corp., Armonk, NY, USA). *P* < 0.05 was considered statistically significant. Concentration–response curves were fitted to the logistic equation using non‐linear regression (PRISM, version 5.0; Graph Pad Software Inc., San Diego, CA, USA). The mean half‐maximal inhibitory concentration of the weak acids (IC_50_) was calculated to compare the inhibitory effects of each weak acid on the amplitude of contraction and AUC.

## Results

### Effect of lactate on spontaneous contractility

Increasing concentrations of sodium lactate (1–20 mM) were applied extracellularly to spontaneously contracting myometrial strips and the effects on contractility weredetermined. In these and subsequent experiments, normal biological variability in contraction parameters can be observed (e.g. in control amplitude). The traces shown throughout are illustrative of the data, and individual traces may differ in some parameters and therefore from the mean data of the group.

**Figure 1:**
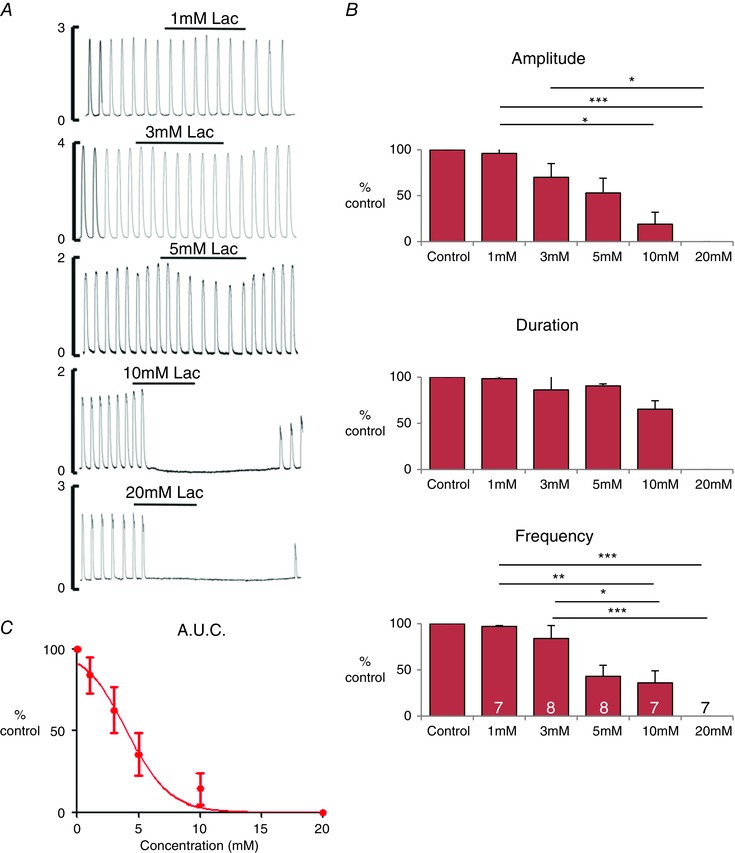
**The effects of lactate on rat myometrium** *A*, typical traces showing the effect of lactate application, from 1 to 20 mM, on spontaneously contracting myometrium. *B*, mean ± SEM data for contraction (i) amplitude, (ii) duration and (iii) frequency. Statistical differences tested by ANOVA are indicated by the asterisks. *P < 0.05. *C*, dose–response curve for contraction AUC (integral of force) and lactate concentration. Recordings were obtained at 35 °C, pH 7.4, in tissue superfused with physiological saline at 2 mL min^–1^.

As shown in Fig. 1 (typical of seven other experiments), the response to lactate was a dose‐dependent decrease in contractility (Fig. 1 *A*). There was a significant decrease in the contractile parameters (i.e. amplitude, frequency and AUC of contractions) compared to the preceding control period (paired Student's *t* test, *P* < 0.05) (Fig. 1 *B*). The amplitude and frequency of contractions was significantly reduced at lactate concentration ≥5 mM. The integral of force were significantly decreased at lactate concentration ≥3 mm. The integral of force was used to plot a dose–response curve as a percentage reduction of force integral compared to control (Fig. 1 *C*). The EC_50_ for the effect of lactate on spontaneously contracting tissue was 3.9±0.3 mM (mean ± sem). As shown in Fig. 1, once lactate was removed and the myometrium returned to PSS, the contractions resumed.

### Oxytocin‐induced contractions

**Figure 2:**
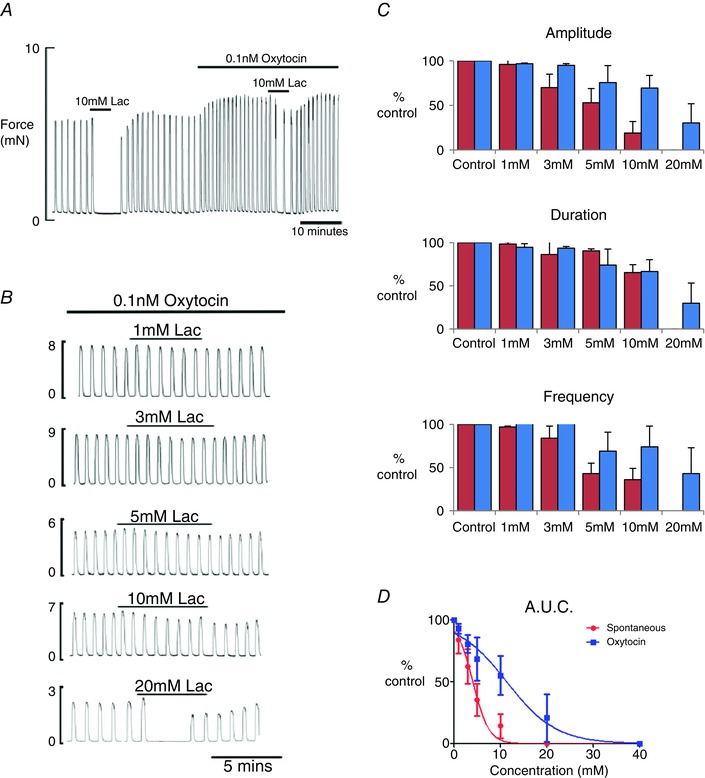
**Effect of oxytocin on the myometrial response to lactate** *A*, application of 10 mm lactate first to spontaneously contracting rat myometrium and then after stimulation with 0.1 nm oxytocin; note the greatly reduced effect of lactate in the presence of oxytocin. *B*, typical traces showing the effect of lactate application, from 1 to 20 mM, on oxytocin stimulated myometrium. *C*, mean ± SEM data showing the effects of lactate in the absence (red; data as presented in Fig. 1) and presence (blue) of oxytocin. *D*, dose–response curves for contraction AUC (integral of force) and lactate concentration in the absence (red) and presence (blue) of oxytocin.

*In vitro* (and *in vivo)* oxytocin stimulates uterine contractility via a variety of mechanisms (Arrowsmith & Wray, 2014). To investigate whether lactate can affect force under such agonist stimulation, the effects of lactate on oxytocin‐induced contractions (*n* = 3–6) were studied (Fig. 2 *A*). Lactate produced a significant decrease in contractility, with all parameters being affected (Fig. 2 *B*) but, as can be seen in comparison to spontaneous activity, these effects were reduced (Fig. 2 *A* and *C*). Thus, there is a significant rightward shift in the dose–response curve in the presence of oxytocin. The EC_50_ for lactate in the presence of oxytocin was calculated to be 11.1 ± 1.7 mM (Fig. 2 *D*).

### Comparative effects of other weak acids

**Figure 3:**
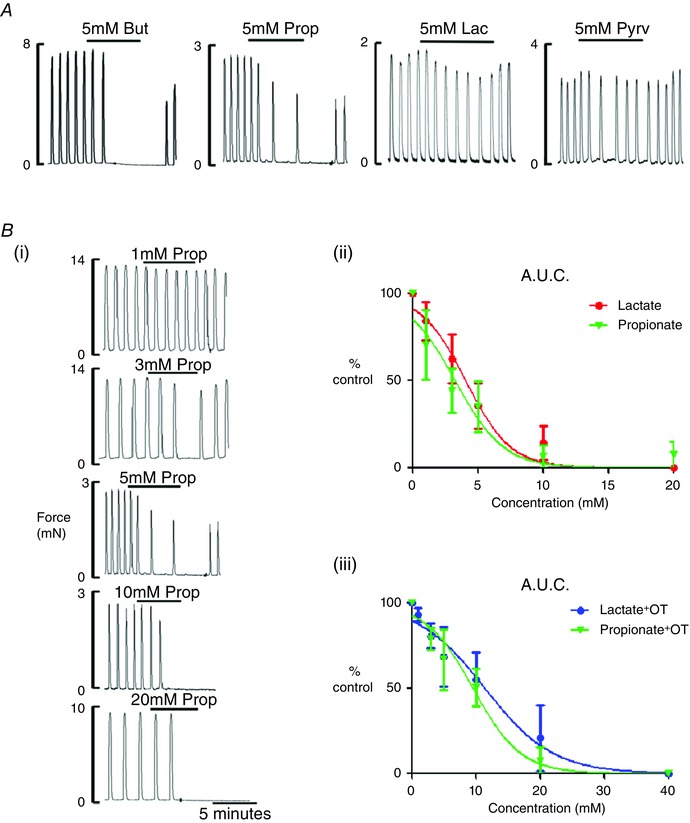
**The effects of weak acids on myometrial contractions** *A*, the effects of 5 mM sodium butyrate, propionate, lactate and pyruvate on spontaneously contacting rat myometrium; typical traces from three to six different preparations. *B*, typical traces (i) showing the effect of propionate application, from 1 to 20 mM, on spontaneously contracting myometrium; dose–response curves (ii) for AUC of spontaneous contractions in the presence of lactate (red) and propionate (green); and dose–response curves (iii) for AUC in the presence of oxytocin, for lactate (red) and propionate (green).

To determine whether the myometrial response to lactate was specific, three other weak acids were tested using the same experimental protocol: butyrate, propionate and pyruvate (*n* = 3–6). The three weak acids were found to produce effects on spontaneous contractions that were very similar to that of lactate: a dose‐dependent decrease in force (Fig. 3 *A*).

Sodium propionate was studied in more detail and its EC_50_ was determined to be 3.2 ± 0.9 mM compared to 3.9 ± 0.3 mM for lactate (Fig. 3 *B*). There was no significant difference between lactate and propionate on spontaneous activity (Fig. 3 *B*ii). Propionate was also tested on oxytocin‐driven activity, where it reduced contractions in all preparations (*n*=6) but, as found for lactate, there was a significant increase in the EC_50_ in the presence of oxytocin to 9.6 ± 1.1 mM (Fig. 3 *B*iii) compared to that found with spontaneous contractions. There was no significant difference in the responses between propionate or lactate on oxytocin‐driven activity.

### Lactate effects on pH_i_ in the myometrium

**Figure 4:**
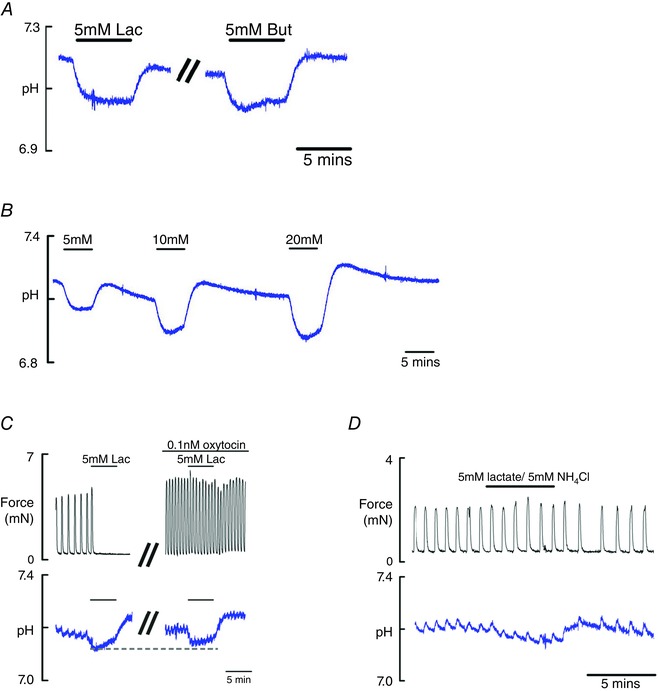
**The effects of lactate on pH_i_ in myometrial smooth muscle** *A*, typical example of the effects of 5 mM sodium lactate and butyrate on pH_i_ measured in the same preparation of rat myometrium. Tissues loaded with carboxy‐SNARF and calibrated using nigericin. *B*, effects of increasing concentrations of lactate on pH_i_. *C*, simultaneous recording of force (top) and pH_i_ (bottom) comparing the effects of 5 mM lactate, in the same preparation, showing its effects on spontaneous and oxytocin stimulated contraction. Note the smaller induced pH change and greatly reduced effect on force when oxytocin is present. *D*, simultaneous application of lactate and NH_4_Cl (5 mM) ‘null’ most of the change in pH and prevent the decrease in force previously shown with lactate application. In (*C*) and (*D*), the notches on the pH traces are a result of the transient changes in pH_i_ that occur with contraction (Taggart & Wray, 1993).

Nothing is known about how lactate may change pH_i_ in the myometrium and how this may relate to effects on contractility. To address these questions, the use of Carboxy‐SNARF in myometrial strips allows the simultaneous measurement of pH_i_ and force to be made. Loading tissue with this indicator did not alter contractile activity, which is consistent with previous findings (Taggart & Wray, 1993). We first tested the effects of the weak base butyrate because this had been previously studied in the myometrium and would therefore provide an appropriate comparison for the lactate measurements. The addition of 5 mM butyrate (Fig. 4 *A*), caused a drop in pH_i_ of 0.13 ± 0.02 pH units, which is consistent with previously reported values (Wray *et al*. 1992). Application of 5 mM lactate to SNARF loaded myometrial strips reduced force and significantly decreased pH_i_ by 0.13 ± 0.01 pH units (*n* = 10; paired Student's *t* test, *P* = 0.002) (Fig. 4 *B*). This was not significantly different from 5 mM butyrate. When lactate was removed, there was a rebound alkalinization and subsequent return of pH_i_ to control values. Spontaneous contractions also reappeared when lactate was removed. Increasing concentrations of lactate caused a significant dose‐dependent decrease in pH_i_ (Fig. 4 *B*): 10 mM caused a drop of 0.19 ± 0.02 pH units (*n *= 5; *P* = 0.001) and 20 mM lactate decreased pH_i_ by 0.24 ± 0.03 pH units (*n *= 5; *P* = 0.001). Lactate (5 mM) was applied in the presence of oxytocin and, as shown in Fig. 4 *C*, this caused a pH drop of 0.09 ± 0.01 pH units (*n* = 4; *P* = 0.001). This was significantly different from the drop produced in spontaneously contracting tissue (unpaired Student's *t* test, *P* = 0.04).

Ammonium chloride is a commonly used weak base that has been previously shown to cause intracellular alkalinization of the myometrium (Parratt *et al*. 1995). To confirm that the acidification produced by lactate is the mechanistical causeof the decrease in uterine force, 5 mM NH_4_Cl was simultaneously applied with 5 mM to ‘null’ the lactate‐induced pH change (Fig. 4 *D*i). The spontaneous contractions continued in the presence of the weak base and acid and pH_i_ fluctuated only slightly. The notches in the pH_i_ records are the normal transient acidification associated with each contractions (Taggart & Wray, 1993).

#### Intracellular Ca^2+^

**Figure 5:**
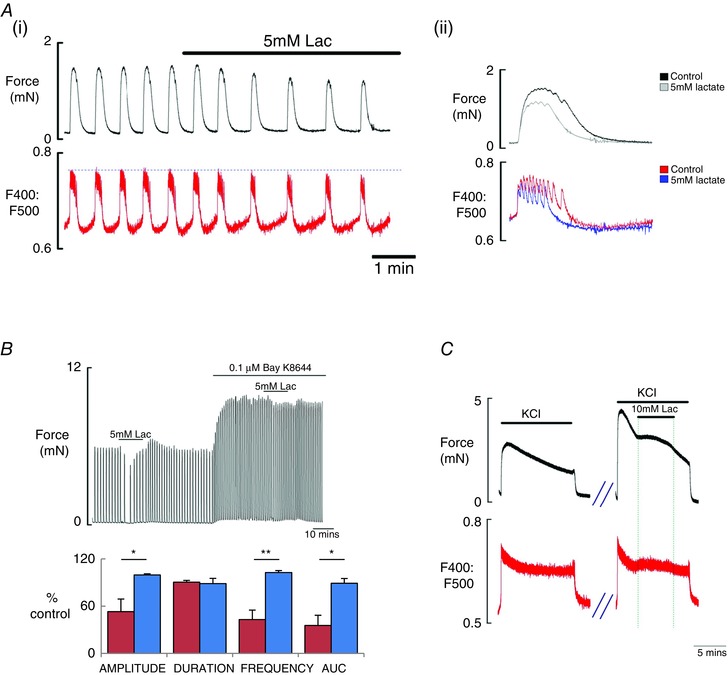
**The effects of lactate on myometrial Ca^2+^ signalling** *A*, simultaneous recording (i) of spontaneous force (top) and Ca^2+^ (Indo‐1 fluorescence, emission signals at 400 and 500 nm) showing the typical effects of lactate (5 mM) application on rat myometrium; inset (ii) is from the trace and expands and overlaps the force and Ca^2+^ records during the control period and during lactate application, to show the changes produced by lactate. *B*, comparison (i) of the effects of lactate using control spontaneous activity and after application of the L‐type Ca^2+^ agonist, Bay K8644 and (ii) comparing mean data and SEM for effects of lactate on contraction amplitude, duration, frequency and AUC, with and without Bay K8644. *C*, the effects of high K^+^ depolarization (40 mM) on force (top) and Ca^2+^ and the effects of lactate on both.

The effect of lactate on intracellular Ca^2+^ signalling was investigated using Indo‐1. In the presence of 5 mM lactate, contractions were decreased as before and the accompanying Ca^2+^ transients decreased (*n *= 7) (Fig. 5A). In four of the seven strips, lactate abolished Ca^2+^ transients and force. In the three strips that continued contracting in the presence of lactate, Ca^2+^ decreased in amplitude (88.8% ± 2.6%). This was mirrored in the force. The Ca^2+^ transients reappeared when lactate was removed with increasing amplitude and this was reflected in the force measurements, which returned to previous control levels. Both the duration and frequency of Ca^2+^ transients were significantly decreased: duration to 79.5 ± 2.0% (*P* = 0.01); frequency to 77.8 ± 0.6% (*P* = 0.01). This can be seen more clearly in the expanded and overlapped portion of the control and lactate traces (Fig. 5*A*ii), where Ca^2+^ spikes are caused by Ca^2+^ entry triggered by individual action potentials, triggering phasic contraction (Burdyga *et al*. 2007; Noble *et al*. 2014). The mean number of spikes present in the transient significantly falls from 20 ± 6 to 13 ± 5 in the presence of lactate (*n* = 3; *P *> 0.05).

To determine whether the effects of lactate were a result of the inhibition of L‐type Ca^2+^ channels, Bay K8644 the Ca^2+^ channel agonist was applied (*n* = 6). In all preparations, application of Bay K8644 stimulated spontaneous contractions, consistent with its ability to increase the opening probability of L‐type Ca^2+^ channels (Yoshino *et al*. 1988). Application of 5 mM lactate in the presence of Bay K8644 is shown in Fig. 5B. The duration of contractions significantly decreased to 92.5 ± 1.9% (paired Student's *t* test, *P* = 0.01) and AUC decreased to 93.9 ± 2.3% (*P* = 0.04). The amplitude and frequency of contractions were not significantly altered (99.7 ± 0.7%, *P* = 0.73; 100.9 ± 2.2%, *P* = 0.69, respectively).

When compared with the effect of lactate on spontaneous activity, using the immediate control period before lactate addition as 100% in both cases, the data show a significant difference in force amplitude and AUC in the presence of lactate with and without Bay K8644 (*n = *6; unpaired Student's *t* test, *P* = 0.03 and *P* = 0.06, respectively)

#### Depolarized preparations

Lactate was applied to KCl (40 mM) depolarized myometrium. As shown in Fig. 5 *C*, application of KCl solution produced a rapid increase in force, which then declined to a plateau throughout the application of solution (*n *= 5). Lactate application during the KCl contraction did not reduce force, and a small but significant increase occurred (118.9 ± 4.5% compared to initial KCl amplitude, 100%; unpaired Student's *t* test, *P* = 0.044). The integral of force (132.4 ± 9.5%) did not reach significance (*P* = 0.54). Force decreased when lactate was removed and returned to basal levels when KCl was removed. Simultaneous force and Ca^2+^ measurements showed a similar small increase in intracellular Ca^2+^ mirrored by increase in force produced by lactate (*n *= 3).

## Discussion

No previous study has investigated the effect of lactate on myometrial contractility or the effect that it will have on pH_i_ and Ca^2+^. Previous work has indicated that lactate plays a role in dysfunctional labour (Quenby *et al*. 2004; Wiberg‐Itzel *et al*. 2014). The present study shows that lactate, and other weak acids, cause a decrease or cessation of contractions in a dose‐dependent manner. Measurement of pH_i_ showed that a modest elevation of lactate will significantly acidify the myometrial cells, although this decrease is tempered in the presence of oxytocin. We also find that the fall in pH is a crucial part of the mechanism by which lactate reduces force because it reduces L‐type Ca^2+^ current and, as is shown in the present study, decreases Ca^2+^ transients arising spontaneously or in the presence of oxytocin.

### The effect of lactate on pH_i_

Given the accumulating data showing that lactate is a key factor in labours characterized by poor contractile activity (Wiberg‐Itzel *et al*. 2011) and the known physiological association of increased lactate production and efflux from exercising or hypoxic myocytes, both of which occur in labour, it is surprising that there are no data exploring the effect of lactate on the myometrium. Previous studies focused on demonstrating that pH_i_ falls with hypoxia in the myometrium and is associated with a decrease in force (Taggart *et al*. 1997; Wray, 2007). From these studies and the *in vivo* data (Larcombe‐McDouall *et al*. 1998), it is clear that decreased pH will be functionally significant in the myometrium. Decreases in pH_i_ as lactate increases have been suggested to explain some (but not all) of the effects of lactate in vascular and cardiac muscles. Effects of lactate independent of pH change have been reported in these tissues and are attributed to effects on membrane potential and, in the heart, reduced conduction velocity, as well as Ca^2+^ handling (Hester *et al*. 1980; Marrannes *et al*. 1981).

That the decrease in force with lactate is largely a result of a fall in pH_i_ is supported by our pH nulling data. When the pH change produced by lactate is neutralized, lactate has no significant effect on force. Thus, the dose‐dependent changes in force produced by lactate are related to its induced size of pH change. This also accounts for its effects being less when oxytocin is present because the pH change is also reduced (in addition to oxytocin also promoting Ca^2+^ entry). However, it is also noted that the induced pH changes were small compared to the large effects on force, suggesting that an additional mechanism may also be present. Thus, in the myometrium, we propose that the induced pH change can account for much of the effect of lactate.

### Effects of weak acids on myometrial contractility

Previous studies have determined how the related weak acid, butyrate, affects pH_i_ and contractions in the myometrium, and have been used to calculate buffering power (Bullock *et al*. 1998). Butyrate was often chosen because it was not expected to have any effect on metabolism, unlike lactate, and therefore provides a clearer data set. We have found that the pH change associated with lactate application to the myometrium is very similar to that of butyrate, as expected from their *K*_d_ values. Furthermore, there is no evidence that processes associated with lactate handling within the cell or its effects on metabolism have any noticeable effect on the pH_i_ change observed. Our data obtained with other weak acids are also consistent with the effect of lactate on the myometrium occurring via changes of pH_i_ because propionate and pyruvic acid also reduced force in a similar way. The EC_50_ values for lactate and propionate of <5 mM indicate that force production in the myometrium is more sensitive than vascular smooth muscle, where values for the EC_50_ value in the range 10–26 mM have been reported (McKinnon *et al*. 1996; Barron & Nair, 2003).

### Oxytocin affects the response to lactate

Of interest was the finding that the pH change elicited by lactate was significantly reduced in the presence of oxytocin. Oxytocin is known to increase contractions by multiple mechanisms, including causing depolarization and Ca^2+^ entry and reducing Ca^2+^ efflux (Arrowsmith & Wray, 2014). Although these factors explain why the decrease in force with acidification is reduced in depolarized or agonist stimulated myometrial preparations, they do not explain why the fall in pH, for the same concentration of lactate, is reduced. Because resting pH is unchanged by oxytocin, a smaller decrease in pH_i_ could be explained if lactate entry into the cell or its dissociation was reduced, or if the increase in protons is better buffered. There is, however, nothing to support such changes in the myometrium. However, the effects of oxytocin on the plasma membrane Ca^2+^‐ATPase, PMCA, may be significant because PMCA counter‐transports protons into the cell (Austin & Wray, 2000; Floyd & Wray, 2007). Under resting conditions, this is not sufficient to alter pH but, in the presence of lactate and depolarization, it may decrease the proton load sufficiently to limit the pH excursion. This reduced acidification, along with the increased stimulus to contraction that oxytocin provides, can explain the reduced effect on force of lactate and suggest that it will mitigate the effects of increasing lactate *in vivo*.

### Effects of lactate on myometrial intracellular Ca^2+^

Previous work by our group on the myometrium has shown that intracellular acidification reduces Ca^2+^ current via L‐type Ca^2+^ channels, the major route of Ca^2+^ entry in myometrium (Shmigol *et al*. 1995; Pierce *et al*. 2003). This produces reduced Ca^2+^ transients and a decreased force of contractions. This probably explains the effects of lactate in our experiments. Consistent with this interpretation are our data showing the simultaneous recordings of force and Ca^2+^, and it is clear that lactate reduced Ca^2+^ entry. Furthermore, if Ca^2+^ entry was stimulated by the L‐type channel agonist BayK8644 or by depolarizing the preparation, force was not reduced by lactate. The inhibition of transients and contractions is reversible and coincides with a return of pH_i_ to resting values when lactate is removed. The Ca^2+^ experiments showed a ‘slow creep’ effect of lactate on the rise to threshold. Making the assumption that Ca^2+^ mirrors membrane potential between contractions, this could be a means by which weak acids affect the frequency of contraction, although this would need direct investigation.

### Lactate range *in vivo*

Our data show the clear effects of lactate on uterine smooth muscle contractions with an EC_50_ of ∼3 mM. This makes the effects found to be within the expected physiological range for extracellular lactate in exercising tissues, especially when blood flow is reduced, as is the case with labouring myometrium (Larcombe‐McDouall *et al*. 1999). Specifically, in myometrial capillary blood from women in labour, the range was 1–5 mmol l^−1^ (Quenby *et al*. 2004), which is consistent with the range over which we found clear effects on force. It is also expected that the concentration will be even higher closer to the cell membrane. Thus, the effects of lactate that we have described are occurring within the physiological range.

### Lactate, hypoxia and smooth muscle force

Hypoxia is known to decrease contraction in myometrial strips and to dilate most systemic blood vessels. Lactate concentration increases during hypoxia and ischaemia because it is the major metabolite formed during anaerobic glycolysis. The effects of lactate on contraction have been studied best in striated muscles, where it is correlated with a decrease in force, although there is still debate about its causative role (Cairns, 2006). Although lactate has been shown to induce relaxation of vascular smooth (Frobert *et al*. 2002), few mechanistic studies have been performed in any visceral smooth muscles, although pH effects on Ca^2+^ and reduced cross‐bridge cycling are suggested (Barron & Nair, 2003; Wendt & Paul, 2003). To the best of our knowledge, these are the first data for the myometrium to show a direct correlation between the effects of increasing lactate and decreased pH_i_ and force of contractions. Thus, lactate will be expected to contribute to the decrease in force found in the myometrium with hypoxia.

### Relation to labour

Lactate changes are closely linked to uterine activity in labour and possible pathophysiological processes. Repeated transient hypoxia is a normal feature of labour because uterine vessels are compressed during contraction (Greiss, 1965; Larcombe‐McDouall *et al*. 1999). With the associated hypoxia and ischaemia comes alteration of metabolites such as ATP, along with a fall in pH and lactate production. The first evidence showing that these normal fluctuations, occurring with each contraction, could become abnormal and problematic in labour was the finding that myometrial capillary blood is not only more acidotic, but also that lactic acidosis occurs in women labouring dysfunctionally (i.e. with weak and unco‐ordinated contractions) (Quenby *et al*. 2004). It was subsequently reported that the increased lactate in such labours could be detected in amniotic fluid (Wiberg‐Itzel *et al*. 2011). This has led to above threshold lactate levels being shown to predict the probability of the need for unplanned Caesarean section (Wiberg‐Itzel *et al*. 2014). Our data now show that the increased lactate in these labours will directly reduce the force of contractions in rat myometrium. Thus, these data provide the first link between the *in vivo* and *in vitro* data on metabolic–contraction coupling studies in the uterus. It remains to be determined, however, whether these effects will be found in human myometrium and if they are affected by gestation. Our data with oxytocin, however, suggest that, in labour, and when its levels increase, there may be a degree of protection from the effects of lactate.

### Summary

Lactate has a direct inhibitory effect on the myometrium from term pregnant rats in the presence and absence of oxytocin. Its affects can be attributed to a decrease in pH_i_ and are dose‐dependent. If pH changes are nulled, then the functional effects of lactate are also removed, suggesting that there are no pH independent effects of lactate on force in the myometrium. The decrease in force is associated with a decrease in Ca^2+^ transients, which can be accounted for by the known effects of pH on myometrial Ca^2+^ current. We suggest that the inhibitory effects of lactate on myometrial contractions contribute to poor contractions in women labouring dysfunctionally when myometrial capillary blood is acidotic as a result of increased lactate.

## Additional information

### Competing interests

There are no conflicts on interest.

### Funding

JH was the recipient of a Wellcome Trust Prize Studentship (Ref. 089141).

### Author contributions

JH, AW and SW designed the experiments. JH performed the experiments and analysed the data. JH, AW and SW wrote the manuscript.
